# Prior AICAR induces elevated glucose uptake concomitant with greater γ3-AMPK activation and reduced membrane cholesterol in skeletal muscle from 26-month-old rats

**DOI:** 10.1139/facets-2021-0166

**Published:** 2022-05-19

**Authors:** Haiyan Wang, Amy Zheng, Edward B. Arias, Gregory D. Cartee

**Affiliations:** a Muscle Biology Laboratory, School of Kinesiology, University of Michigan, Ann Arbor, MI, USA; b Department of Molecular and Integrative Physiology, University of Michigan, Ann Arbor, MI, USA; c Institute of Gerontology, University of Michigan, Ann Arbor, MI, USA

**Keywords:** insulin sensitivity, glucose transport, AMP-activated protein kinase, aging, cholesterol, skeletal muscle

## Abstract

Attenuated skeletal muscle glucose uptake (GU) has been observed with advancing age. It is important to elucidate the mechanisms linked to interventions that oppose this detrimental outcome. Earlier research using young rodents and (or) cultured myocytes reported that treatment with 5-aminoimidazole-4-carboxamide-1-β-d-ribofuranoside (AICAR; an AMP-activated protein kinase (AMPK) activator) can increase γ3-AMPK activity and reduce membrane cholesterol content, each of which has been proposed to elevate GU. However, the effect of AICAR treatment on γ3-AMPK activity and membrane cholesterol in skeletal muscle of aged animals has not been reported. Our purpose was to evaluate the effects of AICAR treatment on these potential mechanisms for enhanced glucose uptake in the skeletal muscle of aged animals. Epitrochlearis muscles from 26–27-month-old male rats were isolated and incubated ± AICAR, followed by 3 h incubation without AICAR, and then incubation with 3-*O*-methyl-[^3^ H] glucose (to assess GU ± insulin). Muscles were also analyzed for γ3-AMPK activity and membrane cholesterol content. Prior AICAR treatment led to increased γ3-AMPK activity, reduced membrane cholesterol content, and enhanced glucose uptake in skeletal muscle from aged rats. These observations revealed that two potential mechanisms for greater GU previously observed in younger animals and (or) cell models are also potentially relevant for enhanced GU by muscles from older animals.

## Introduction

Epidemiological data indicate that the prevalence of prediabetes and diabetes is progressively increased with advancing age (Cowie et al. 2009). Skeletal muscle is the tissue responsible for the major portion of insulin-mediated glucose disposal, and insulin resistance is an essential defect contributing to the development of type 2 diabetes (DeFronzo et al. 2015). These circumstances motivate efforts to identify and understand interventions that improve glucose uptake by skeletal muscle at advanced ages.

Previous exercise research has demonstrated that skeletal muscle retains a considerable reserve capacity to increase muscle glucose uptake even during old age (Cartee et al. 1993; Xiao et al. 2013; Sharma et al. 2015; Cartee et al. 2016; Oki et al. 2020). However, not all older individuals have the ability and (or) willingness to perform sufficient exercise to gain this important health benefit. Accordingly, it is worthwhile to explore other strategies to enhance muscle glucose uptake during advanced age. Strong evidence links the activation of skeletal muscle AMPK (adenosine monophosphate-activated protein kinase) to elevated glucose uptake by skeletal muscle. Fisher et al. (2002) reported that the incubation of isolated rat skeletal muscle with the AMPK activator AICAR (5-aminoimidazole-4-carboxamide-1-β-d-ribofuranoside) results in subsequently increased insulin-stimulated glucose uptake. AMPK is a heterodimeric enzyme that is comprised of alpha (α1 or α2), beta (β1 or β2), and gamma (γ1, γ2, or γ3) subunits (Kjobsted et al. 2016; Kjobsted et al. 2018). The γ3 subunit is notable because it is almost exclusively expressed in skeletal muscle, and the ability of AICAR-treatment to enhance insulin-stimulated glucose uptake by rodent skeletal muscle is absent in γ3-knockout (KO) mice (Mahlapuu et al. 2004; Kjobsted et al. 2015). These results provide compelling evidence that γ3-AMPK is essential for this important outcome.

The enhanced insulin-stimulated glucose uptake in skeletal muscle several hours after AICAR treatment is accompanied by greater phosphorylation of a protein known as AS160 (also called Akt substrate of 160 kDa or TBC1D4) (Kjobsted et al. 2015). AS160 is a Rab-GTPase activating protein that plays a key role in regulating insulin-stimulated GLUT4 glucose transporter translocation and glucose uptake (Sano et al. 2003; Cartee 2015). Prior AICAR treatment leads to greater AS160 phosphorylation on key phosphosites that regulate insulin-stimulated glucose uptake, and the improvement in insulin-stimulated glucose uptake after AICAR treatment is not found in the skeletal muscle of AS160-KO mice (Kjobsted et al. 2019). These observations implicate AS160 as a key protein for AICAR-induced improvement in glucose uptake.

We recently reported that prior treatment of skeletal muscle from old rats with AICAR resulted in increased insulin-stimulated glucose uptake that was accompanied by greater AS160 phosphorylation (Oki et al. 2018). However, the effect of AICAR on γ3-AMPK activity in the skeletal muscle of aged animals has not been previously reported. Therefore, our first aim was to determine if AICAR leads to greater γ3-AMPK activity in skeletal muscle from old rats.

In addition to the evidence that AMPK-induced phosphorylation of AS160 is important for the elevated insulin-stimulated glucose uptake in skeletal muscle after AICAR treatment, there is also support for another possible mechanism to contribute to the AICAR/AMPK-dependent increase in glucose uptake. An inverse relationship has been observed between membrane cholesterol and glucose uptake by skeletal muscle (Grice et al. 2019; Habegger et al. 2012b; Sanchez-Aguilera et al. 2018). Furthermore, research using L6 skeletal muscle cells indicated that AICAR can stimulate AMPK, lower membrane cholesterol, and increase insulin-stimulated glucose uptake (Habegger et al. 2012a). Therefore, the second aim was to determine if AICAR treatment of skeletal muscle from old rats resulted in altered membrane cholesterol content. HMGCR (3-hydroxy-3-methylglutaryl coenzyme A reductase), the rate-limiting enzyme for cholesterol synthesis, is phosphorylated by AMPK on a site that regulates HMGCR activity (Clarke and Hardie 1990), and ABCA1 (ATP-binding cassette transporter A1) is an important protein for cellular cholesterol efflux (Wang and Tall 2003; Larrede et al. 2009). Accordingly, we also evaluated the effect of AICAR treatment on the phosphorylation of HMGCR and ABCA1 in the skeletal muscle of old rats.

TBC1D1 is an AS160-paralog and Rab-GTPase-activating protein expressed by skeletal muscle that can be phosphorylated by AMPK in response to AICAR treatment and that regulates glucose uptake (Taylor et al. 2008; Cartee 2015; Chen et al. 2017). However, the effect of prior AICAR treatment on TBC1D1 phosphorylation in skeletal muscle of old rats has not been previously reported.

Accordingly, our third aim was to determine prior AICAR treatment’s effect on phosphorylation of TBC1D1 on Ser237, a site implicated in AICAR-stimulated glucose uptake (Taylor et al. 2008; Chen et al. 2017). We also evaluated TBC1D1 Thr590, a site that can be phosphorylated in response to insulin (Pehmoller et al. 2009; Vichaiwong et al. 2010).

## Materials and methods

### Materials

Chemicals were purchased from Sigma-Aldrich (St. Louis, MO) or Fisher Scientific (Hanover Park, IL) unless otherwise noted. The reagents and apparatus for SDS-PAGE and nonfat dry milk (no. 170-6404) were obtained from Bio-Rad (Hercules, CA). Pierce MemCode Reversible Protein Stain Kit (#24585), Bicinchoninic acid protein assay (#23225), Tissue Protein Extraction Reagent (TPER; #78510), Protein G magnetic beads (#10004D), and DynaMag^™^-2 magnet (#12321D) were from Thermo Fisher Scientific (Waltham, MA). Anti-phospho Akt Ser^473^ (pAkt^Ser473^; #9271), anti-phospho Akt Thr^308^ (pAkt^Thr308^; #13038), anti-Akt (#4691), anti-phospho AS160 Thr^642^ (pAS160^Thr642^; #8881), anti-phospho AS160 Ser^588^ (pAS160^Ser588^; #8730), anti-phospho AMPKα Thr^172^ (pAMPKa^Thr172^; #2531), anti-AMP-activated protein kinase-α (AMPKα; #5831), anti-acetyl CoA carboxylase (ACC; #3676), anti-phospho ACC Ser79 (pACC^Ser79^; #3661), anti-TBC1D1 (#5929), anti-hexokinase II (HKII; #2867), anti-insulin receptor (IR; #3025), anti-α-Tubulin (#2144), anti-Na^+^/K^+^-ATPase (#3010) and anti-rabbit IgG horseradish peroxidase conjugate (#7074) were from Cell Signaling Technology (Danvers, MA). Anti-phospho TBC1D1 Thr590 (pTBC1D1^Thr590^; #AF2422) was from Sapphire North America (Ann Arbor, MI). Anti-phospho AS160 Ser^704^ (pAS160^Ser704^) was provided by Dr. Jonas Thue Treebak (Novo Nordisk Foundation Center for Basic Metabolic Research, University of Copenhagen, Denmark). Anti AMP-activated protein kinase γ3 (γ3-AMPK) was provided by Dr. David Thomson (Brigham Young University, USA) (Hardman et al. 2014). Anti-Akt Substrate of 160 kDa (AS160; #ABS54), Anti-GLUT4 (GLUT4; #CBL243), Anti-phospho-TBC1D1 Ser237 (pTBC1D1^Ser237^; #07-2268), P81 Phosphocellulose Squares (#20-134) and enhanced chemiluminescence Luminata Forte Western HRP Substrate (#WBLUF0100) were from MilliporeSigma (Billerica, MA). Anti-HMGCR (HMGCR, #BS-5068 R) and anti-phospho HMGCR Ser872 (pHMGCR^Ser872^, # BS-4063 R) were from Bioss Antibodies (Woburn, MA). Anti-ABCA1 (ABCA1, #NB400-105SS) was from Novus Biologicals (Littleton, CO). Anti-phospho ABCA1 Ser2054 (pABCA1^Ser2054^; #ab12064) was from Abcam (Cambridge, MA). 3-*O*-methyl-[^3^ H] glucose ([^3^ H]3-MG) was from Sigma-Aldrich, and [^14^C] mannitol was from PerkinElmer (Boston, MA). [γ-^33^ P]-ATP was from American Radiolabeled Chemicals, Inc. (St. Louis, MO). Liquid scintillation cocktail (#111195-CS) was from Research Products International (Mount Prospect, IL).

### Animal treatment

Animal care procedures were approved by the University of Michigan Committee on Use and Care of Animals and performed in accordance with the guidelines from the Guide for the Care and Use of Laboratory Animals of the National Institutes of Health, USA. Male Fischer-344 X Brown Norway rats were obtained from the National Institute of Aging (NIA) rodent colony at approximately 22–23 months old. Rats were individually housed at the University of Michigan (12:12 h light:dark cycle, lights out at 17:00 h), provided with standard rodent chow (Laboratory Diet no. 5L0D; LabDiet, St. Louis, MO) and water ad libitum. The terminal experiment was performed when the rats were approximately 26–27 months old, and the rats were fasted at approximately 17:00 on the night before the experiment.

### Muscle dissection and incubation

Rats were deeply anesthetized by an intraperitoneal injection of ketamine–xylazine cocktail (50 mg/kg ketamine and 5 mg/kg xylazine), and both epitrochlearis muscles from each rat were dissected out and longitudinally split into two muscle strips. The muscle strips were placed in vials that were shaken at 45 oscillations per minute and continuously gassed (95% O_2_/5% CO_2_) in a heated (35 °C) water bath. For muscles analyzed to determine 3-MG uptake and signaling proteins, each muscle strip was incubated with a four-step process (Incubation Protocol 1). During step 1 (60 min), muscle strips were incubated with Krebs-Henseleit Buffer (KHB) supplemented with 8 mM glucose ± 2 mM AICAR (2 mM mannitol for without AICAR). During step 2 (180 min), all muscle strips were incubated in KHB supplemented with 8 mM glucose and 2 mM mannitol in the absence of AICAR. During step 3 (30 min), muscle strips were incubated with KHB supplemented with 0.1% bovine serum albumin (BSA), 2 mM sodium pyruvate, and 6 mM mannitol ± insulin (1.2 nM). During step 4 (20 min), muscle strips were incubated with KHB–BSA, the same concentration of insulin as previous step 3, 8 mM 3-MG (final specific activity of 250 μCi/mmol, [^3^ H]3-MG), and 2 mM mannitol (final specific activity of 50 μCi/mmol [^14^C]-mannitol). After step 4, the final incubation step, muscles were blotted, freeze-clamped, and stored at −80 °C for later processing and analysis.

For muscles analyzed to determine γ3-AMPK activity, two muscle strips from one epitrochlearis muscle were incubated with KHB ± 2 mM AICAR (2 mM mannitol for without AICAR) for 60 min (Incubation Protocol 2), then the muscle strips were blotted, freeze-clamped, and stored at −80 °C for later analysis. Two muscle strips from the contralateral epitrochlearis muscle were incubated with a four-step process (Incubation Protocol 3) in which step 1 and step 2 were the same as Incubation Protocol 1. During steps 3 and 4 (30 min and 20 min), muscle strips were incubated with KHB supplemented with 0.1% BSA, 2 mM sodium pyruvate and 6 mM mannitol with insulin (1.2 nM). For muscles analyzed to determine membrane cholesterol and cholesterol regulatory proteins, each muscle strip from both epitrochlearis muscles was incubated with Incubation Protocol 3. After the final incubation step, muscles were blotted, freeze-clamped, and stored at −80 °C until later processing and analysis.

### Muscle lysate preparation

Frozen muscles were weighed and homogenized with 1 mL ice-cold lysis buffer using a glass pestle attached to motorized homogenizer (Caframo, Georgian Bluffs, ON). For muscle lysates analyzed to determine 3-MG uptake, protein abundance and phosphorylation by immunoblotting, the lysis buffer contained T-PER Tissue Protein Extraction Reagent (#PI-78510; Thermo Scientific, Rockford, IL) supplemented with 1 mM EDTA, 1 mM EGTA, 2.5 mM sodium pyrophosphate (NaPPi), 1 mM sodium orthovanadate (Na_3_VO_4_), 1 mM ß-glycerophosphate, 1 μg/mL leupeptin, and 1 mM phenylmethylsulfonyl fluoride (PMSF). For muscle lysates analyzed to determine γ3-AMPK activity, the lysis buffer contained 10% glycerol, 20 mM NaPPi, 1% NP-40, 2 mM PMSF, 150 mM sodium chloride (NaCl), 50 mM HEPES (pH 7.5), 20 mM β-glycerophosphate, 10 mM sodium fluoride (NaF), 1 mM EDTA, 1 mM EGTA, 10 μg/mL aprotinin, 10 μg/mL leupeptin, and 2 m MNa_3_VO_4_. Homogenates were rotated for 1 h at 4 °C prior to centrifugation (15,000 g for 15 min at 4 °C). The supernatants were transferred to microfuge tubes and stored at −80 °C until subsequent analyses. Protein concentration was measured using the bicinchoninic acid procedure.

### 3-MG uptake

Aliquots of the supernatants (200 μL) from muscle lysates were pipetted into a vial together with scintillation cocktail. A scintillation counter (PerkinElmer) was used to determine the ^3^H and ^14^C disintegrations per minute. 3-MG uptake was calculated as described by Cartee and Bohn (1995).

### γ3-AMPK activity assay

The specificity of the γ3-AMPK antibody used for immunoprecipitation (IP) was previously confirmed in Wang et al. (2018). AMPK activity was determined as described by Kjobsted et al. (2015). Briefly, muscle lysates (300 μg protein) were rotated with antibody γ3-AMPK (1:500) and IP buffer [50 mM NaCl, 1% Triton X-100, 50 mM NaF, 5 mM NaPPi, 20 mM Tris-base (pH 7.5), 500 μM PMSF, 2 mM dithiothreitol (DTT), 5 μg/mL leupeptin, 50 μg/mL soybean trypsin inhibitor, 6 mM benzamidine, and 250 mM sucrose] at 4 °C overnight. 50 μL of protein G-magnetic beads were added to each sample, then the samples were rotated for 2 h at 4 °C. DynaMag^™^-2 magnet was used to pellet the protein G-immunocomplex. Each immunopellet was washed once in IP buffer, once in 6 × assay buffer (240 mM HEPES, 480 mM NaCl, pH 7.0), twice in 3 × assay buffer (1:1). The reaction was initiated at 30 °C by the addition of 30 μL of kinase mix buffer (40 mM HEPES, pH 7.5, 80 mM NaCl, 800 μM DTT, 200 μM AMP, 100 μM AMARA peptide, 5 mM magnesium chloride, 200 μM ATP, and 2 μCi of [γ-^33^ P]-ATP). After 30 min, the reaction was stopped by the addition of 10 μL of 1% phosphoric acid. Next, 30 μL of supernatant was spotted on P81 phosphocellulose paper. After 3 × 15 min washing with 1% phosphoric acid, followed by 1 × 5 min washing with acetone, the phosphocellulose paper was dried at room temperature and placed in the vials containing 8 mL scintillation cocktail for scintillation counting. Results were expressed relative to the normalized mean of all the samples from each experiment.

### Membrane fractionation and cholesterol content measurement

Membrane-enriched and cytosol-depleted fractions were obtained by differential centrifugation as described by Grice et al. (2019). Briefly, epitrochlearis muscles were homogenized in ice-cold HES buffer (20 mM HEPES, pH 7.4, 2 mM EGTA, and 250 mM sucrose, 200 μM PMSF, 10 μg/mL pepstatin, and 1 μg/mL leupeptin) with a Polytron PT-3100 homogenizer. The homogenates were centrifuged at 1,380 g for 30 min at 4 °C, and the resulting supernatant was saved. The pellet was resuspended with HES buffer and centrifuged at 1,380 g for 30 min at 4 °C, the resulting supernatant was saved. Then the two supernatants were combined and centrifuged at 17,000 g for 30 min at 4 °C. This combined supernatant (cytosol fraction) was saved. The pellet was washed with HES buffer and centrifuged at 1,380 g for 30 min at 4 °C, the resulting supernatant was saved. Then this supernatant was centrifuged at 17,000 g for 30 min at 4 °C. The resulting pellet (membrane-enriched fraction) was resuspended in HES buffer and stored at −80 °C until analysis.

Membrane enrichment was assessed by immunoblotting with antibodies against membrane marker proteins (insulin receptor, IR; Na^+^/K^+^-ATPase) and a cytosolic marker protein (α-tubulin). Cholesterol content in the membrane-enriched fraction was determined using the Amplex Red Cholesterol Assay Kit (Thermo Scientific; #A12216) as described by Grice et al. (2019).

### Immunoblotting

Immunoblotting procedures were described by Wang et al. (2018). An equal amount of protein from each muscle lysate was mixed with 6 × Laemmli buffer, boiled for 5 min, separated using SDS-PAGE, and then transferred to polyvinylidene difluoride membranes. Equal loading was confirmed using the MemCode protein stain (Antharavally et al. 2004). Membranes were blocked with TBST (Tris-buffered saline, pH 7.5 plus 0.1% Tween-20) that was supplemented with either with 5% BSA or 5% nonfat milk for 1 h at room temperature, incubated with appropriate concentrations of primary and secondary antibodies. Then membranes were subjected to enhanced chemiluminescence and quantified by densitometry (AlphaView; ProteinSimple, San Jose, CA). Results for each sample (densitometric units) were expressed relative to the normalized average of all the samples on the blot. These normalized values were divided by the corresponding MemCode loading control value for each sample (using individual sample MemCode values that were normalized by dividing the mean MemCode values for all samples on each blot). Values for phosphorylated proteins were expressed as the ratio of phosphorylated signaling protein or enzyme to total signaling protein or enzyme (determined for each sample using a separate immunoblot with a primary antibody against the appropriate total signaling protein or enzyme).

### Statistical analysis

Student’s t-test was used for comparisons between two groups. Two-way analysis of variance (ANOVA) was used to identify main effects of insulin (0 or 1.2 nM insulin) and AICAR (0 or 2 mM AICAR). Post-hoc analysis was performed using the Tukey test (SigmaPlot version 14.5; Systat Software, San Jose, CA). Data lacking normal distribution and (or) equal variance were mathematically transformed to achieve normality and equal variance prior to statistical analysis.

## Results

### 3-MG uptake

**Fig. 1.**
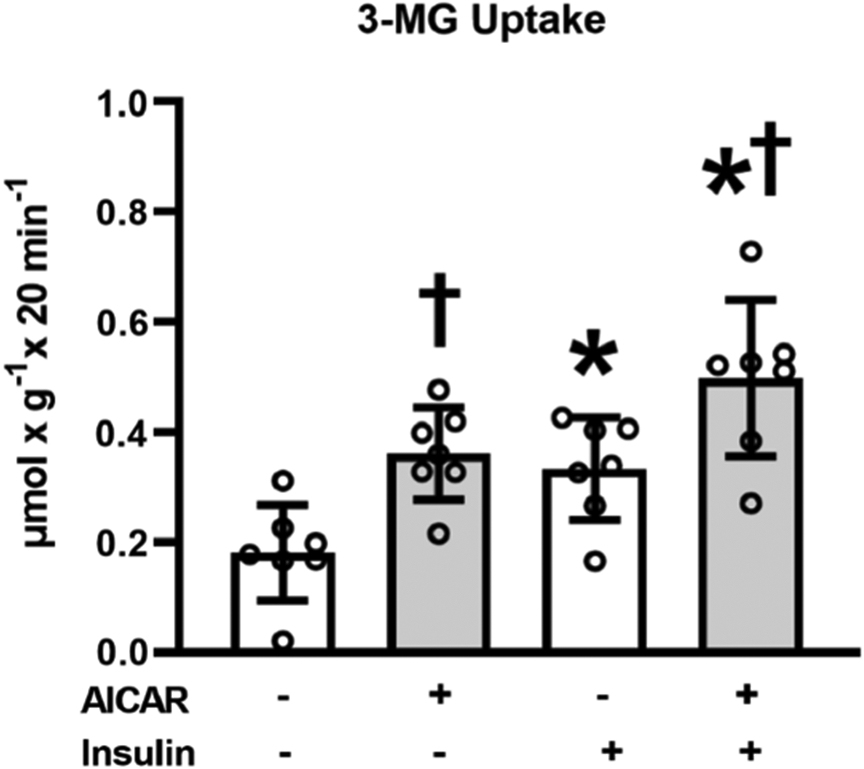
3-O-methyl-glucose (3-MG) uptake in epitrochlearis muscles. *Insulin versus without insulin within the same 5-aminoimidazole-4-carboxamide-1-*β*-d-ribofuranoside (AICAR) treatment (*P* < 0.05). ^†^AICAR versus without AICAR within the same insulin concentration (*P* < 0.01). Data were analyzed using two-way analysis (insulin × AICAR) of variance. Tukey post hoc analysis was performed to identify significant differences. Values are means ± SD; *n* = 7 per treatment group.

Glucose uptake with insulin alone exceeded values without either AICAR or insulin (*P* < 0.05; Fig. 1), and glucose uptake in the AICAR + insulin group exceeded both the insulin alone group (*P* < 0.01) and the AICAR alone group (*P* < 0.05).

### γ3-AMPK activity

**Fig. 2.**
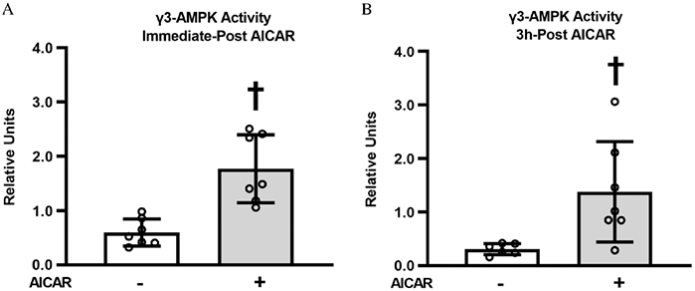
(A) γ3-AMPK activity in epitrochlearis muscles from immediately post 5-aminoimidazole-4-carboxamide-1-*β*-d-ribofuranoside (AICAR) treatment. (B) γ3-AMPK activity in epitrochlearis muscles from 3 h post AICAR treatment. ^†^AICAR versus without AICAR at the same time point (*P* < 0.001 for immediate post AICAR; *P* < 0.01 for 3 h post AICAR). Data were analyzed by Student’s *t*-test, Values are means ± SD; *n* = 6–7 per treatment group. Immediately-post AICAR, immediately post AICAR treatment; 3 h-Post AICAR, 3 h post AICAR treatment. Note: AMP-activated protein kinase, AMPK.

To gain insight into the time-course for AICAR effects on γ3-AMPK activity, we determined γ3-AMPK activity immediately post-AICAR treatment (immediate post-AICAR) and 3 h post-AICAR treatment (3 h post-AICAR). γ3-AMPK activity was increased immediate post-AICAR (*P* < 0.001, Fig. 2A). After 3 h of recovery from AICAR stimulation, muscles with AICAR treatment had greater γ3-AMPK activity compared with unstimulated control muscles (*P* < 0.01, Fig. 2B). This result indicates a persistent effect of prior AICAR stimulation on γ3-AMPK activity.

### Immunoblotting

For all of the phosphorylated proteins, the data were expressed as a ratio of the phosphorylated to total protein values.

### Total abundance of signaling proteins

There were no significant effects of insulin or AICAR on total Akt, AMPK, and TBC1D1 abundance. Total AS160 abundance in muscles without insulin was less than in muscles with insulin, either without (~35%, *P* < 0.05) or with prior AICAR treatment (~29%, *P* < 0.05). ANOVA revealed a significant Insulin × AICAR interaction for total ACC abundance (*P* < 0.05). Total ACC abundance in the AICAR alone group was less (~22%, *P* < 0.01) than in the group without either AICAR or insulin (*P* < 0.01).

### Akt phosphorylation

**Fig. 3.**
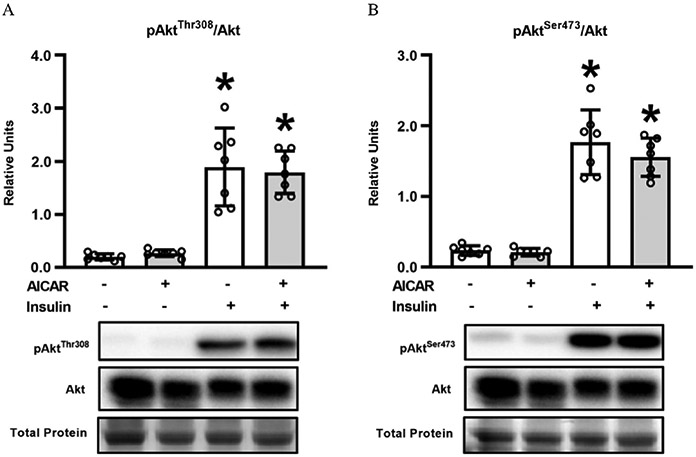
(A) Phosphorylated Akt^Thr308^/Akt and (B) Phosphorylated Akt^Ser473^/Akt, in epitrochlearis muscles. *Insulin versus without insulin with the same 5-aminoimidazole-4-carboxamide-1-*β*-d-ribofuranoside (AICAR) treatment (*P* < 0.001 for pAkt^Thr308^/Akt and pAkt ^Ser473^/Akt). Data were analyzed using two-way analysis (insulin × AICAR) of variance. Tukey post hoc analysis was performed to identify significant differences. Values are means ± SD; *n* = 7 per treatment group. The figure includes representative blots of phosphorylated signaling proteins and corresponding total signaling proteins below the graph. Total protein (based on MemCode staining) served as the loading control.

Akt^Thr308^ phosphorylation in muscles with insulin was greater than in muscles without either AICAR or insulin (*P* < 0.001; Fig. 3A), as well as insulin + AICAR exceeded AICAR alone (*P* < 0.001). Akt^Ser473^ phosphorylation was increased by insulin, either in the absence (*P* < 0.001; Fig. 3B) or presence of AICAR (*P* < 0.001).

### AS160 phosphorylation

**Fig. 4.**
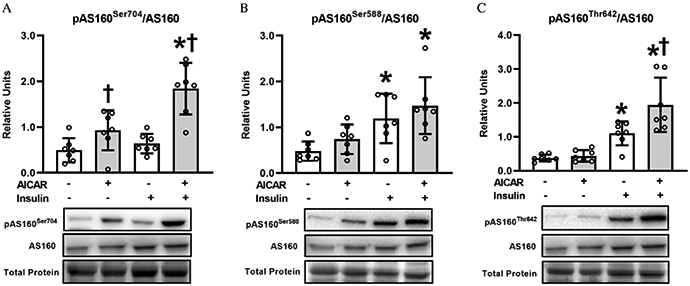
(A) Phosphorylated AS160^Ser704^/AS160; (B) Phosphorylated AS160 ^Ser588^/AS160 and (C) Phosphorylated AS160^Thr642^/AS160, in epitrochlearis muscles. *Insulin versus without insulin within the same 5-aminoimidazole-4-carboxamide-1-*β*-d-ribofuranoside (AICAR) treatment (*P* < 0.01 with AICAR for pAS160^Ser704^/AS160; *P* < 0.01 for pAS160^Ser588^/AS160, *P* < 0.001 for pAS160^Thr642^/AS160). ^†^AICAR versus without AICAR with the same insulin concentration (*P* < 0.05 without insulin and *P* < 0.001 with insulin for pAS160^Ser704^/AS160; *P* < 0.01 with insulin for pAS160^Thr642^/AS160). Data were analyzed using two-way analysis (insulin × AICAR) of variance. Tukey post hoc analysis was performed to identify significant differences. Values are means ± SD; *n* = 7 per treatment group. The figure includes representative blots of phosphorylated signaling proteins and corresponding total signaling proteins below the graph. Total protein (based on MemCode staining) served as the loading control. Note: Akt substrate of 160 kDa, AS160.

ANOVA revealed a significant Insulin × AICAR interaction for AS160^Ser704^ phosphorylation (*P* < 0.05; Fig. 4A) and AS160^Thr642^ phosphorylation (*P* < 0.05; Fig. 4C). AS160^Ser704^ phosphorylation with AICAR alone exceeded without AICAR or insulin (*P* < 0.05; Fig. 4A), and the AICAR + insulin group was greater than both the insulin alone group (*P* < 0.001) and the AICAR alone group (*P* < 0.001). AS160^Ser588^ phosphorylation was increased by insulin, either in the absence (*P* < 0.01; Fig. 4B) or presence of AICAR (*P* < 0.01). AS160^Thr642^ phosphorylation with insulin alone was greater than without either AICAR or insulin, and the AICAR + insulin group exceeded both the insulin alone group (*P* < 0.01; Fig. 4C) and the AICAR alone group (*P* < 0.001).

### AMPKα and ACC phosphorylation

**Fig. 5.**
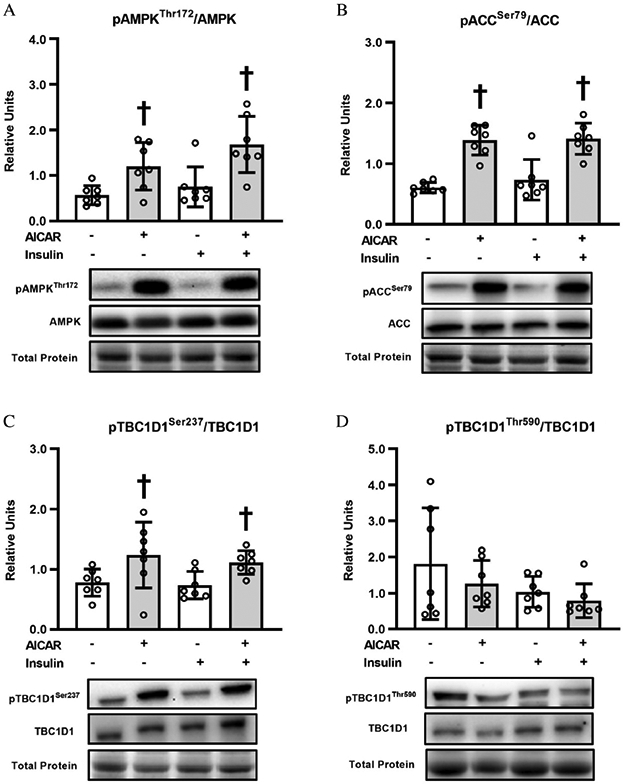
(A) Phosphorylated AMPKα^Thr172^/AMPKα; (B) Phosphorylated ACC^Ser79^/ACC; (C) Phosphorylated TBC1D1^Ser237^/TBC1D1; (D) Phosphorylated TBC1D1^Thr590^/TBC1D1, in epitrochlearis muscles. ^†^5-aminoimidazole-4-carboxamide-1-*β*-d-ribofuranoside (AICAR) versus without AICAR with the same insulin concentration (*P* < 0.05 without insulin and *P* < 0.01 with insulin for pAMPKα^Thr172^/AMPKα; *P* < 0.001 for pACC^Ser79^/ACC; *P* < 0.05 for pTBC1D1^Ser237^/TBC1D1). Data were analyzed using two-way analysis (insulin × AICAR) of variance. Tukey post hoc analysis was performed to identify significant differences. Values are means ± SD; *n* = 7 per treatment group. The figure includes representative blots of phosphorylated signaling proteins and corresponding total signaling proteins below the graph. Total protein (based on MemCode staining) served as the loading control. Note: AMP-activated protein kinase, AMPK; acetyl CoA carboxylase, ACC.

AMPKα^Thr172^ (Fig. 5A) or ACC^Ser79^ phosphorylation (Fig. 5B) was increased by prior AICAR treatment, regardless of insulin concentration (*P* < 0.05 without insulin; *P* < 0.01 with insulin for pAMPKα^Thr172^; *P* < 0.001 without or with insulin for pACC^Ser79^).

### TBC1D1 phosphorylation

TBC1D1^Ser237^ phosphorylation was increased with prior AICAR treatment, either in the absence (*P* < 0.05; Fig. 5C) or presence of insulin (*P* < 0.05). For TBC1D1^Thr590^ phosphorylation, there were no significant effects of insulin or AICAR (Fig. 5D).

### GLUT4 and HKII abundance

**Fig. 6.**
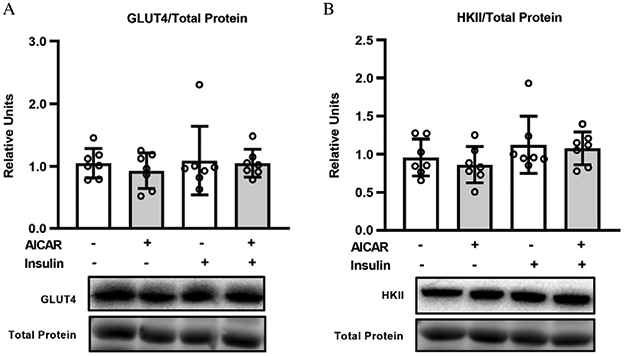
(A) GLUT4 abundance in epitrochlearis muscles. (B) Hexokinase II abundance in epitrochlearis muscles. Data were analyzed using two-way analysis (insulin × 5-aminoimidazole-4-carboxamide-1-*β*-d-ribofuranoside (AICAR)) of variance. Tukey post hoc analysis was performed to identify significant differences. Values are means ± SD; *n* = 7 per treatment group. The figure includes representative blots. Total protein (based on MemCode staining) served as the loading control.

There were no significant effects of insulin or AICAR on either GLUT4 or HKII abundance. (Fig. 6A and 6B).

### Membrane cholesterol content

**Fig. 7.**
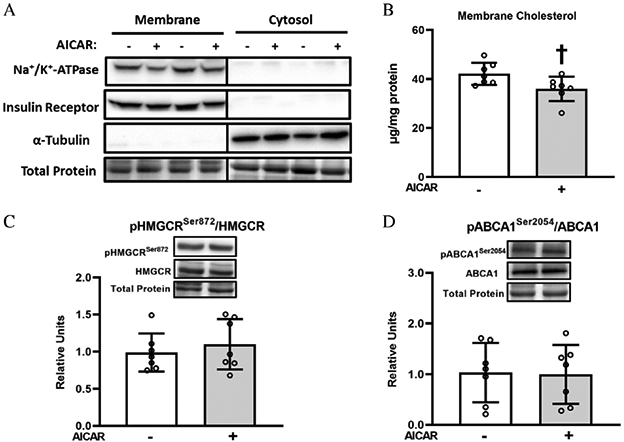
(A) Membrane and cytosolic marker proteins. (B) Membrane cholesterol content, (C) pHMGCR^Ser872^/HMGCR and (D) pABCA1^Ser2054^/ABCA1, in epitrochlearis muscles. ^†^5-aminoimidazole-4-carboxamide-1-*β*-d-ribofuranoside (AICAR) versus without AICAR, *P* < 0.05. Data were analyzed by Student’s *t*-test, values are means ± SD; *n* = 6–7 per group. The figure includes representative blots of phosphorylated enzymes and corresponding total enzymes above the graph. Total protein (based on MemCode staining) served as the loading control. Lysates for membrane and cytosol enriched fractions were loaded on the same gel. The vertical line between the final lane for the membrane samples and the initial lane for the cytosol samples denotes that the membrane and cytosol samples were loaded on the same gel. The individual membrane samples and cytosol samples were from the same muscles. Note: 3-hydroxy-3-methylglutaryl coenzyme A reductase, HMCR; ATP-binding cassette transporter A1, ABCA1.

The membrane fraction was enriched with membrane marker proteins (IR, Na^+^/K^+^-ATPase) and depleted of the cytosolic marker protein (α-tubulin; Fig. 7A). Skeletal muscle membrane cholesterol content was decreased by prior AICAR treatment (*P* < 0.05; Fig. 7B).

### HMGCR and ABCA1 phosphorylation

Phosphorylated HMGCR^Ser872^ and phosphorylated ABCA1^Ser2054^ were unaltered by prior AICAR treatment (Fig. 7C and 7D).

## Discussion

The purpose of the current study was to evaluate several potential mechanisms that might contribute to the AICAR-induced enhancement of glucose uptake by skeletal muscle from 26–27-month-old rats. The results revealed that prior AICAR treatment of skeletal muscle resulted in a robust and sustained increase in γ3-AMPK activity. Prior AICAR treatment also resulted in a significant decline in membrane cholesterol content. In addition, AICAR-treated muscle had greater TBC1D1^Ser237^ phosphorylation. Based on these results, it is possible that one or more of these outcomes contributed to the AICAR-related increase in glucose uptake.

Muscles incubated with AICAR had greater glucose uptake determined both in the presence and absence of insulin. We previously used the same incubation protocol with muscles from 24-month-old, male Fischer-344 X Brown Norway (FBN) rats, and found that prior AICAR treatment led to increased glucose uptake in muscles incubated with insulin, but not in muscles incubated without insulin (Oki et al. 2018). Both the current study and Oki et al. (2018) evaluated isolated epitrochlearis muscles from male FBN rats. It is uncertain if the modest difference in age (26–27-month-old in current study versus 24-month-old in Oki et al. (2018)) was a factor in the differing effects of AICAR on insulin-independent glucose uptake. However, the relative AICAR-induced increase in glucose in insulin-stimulated muscles was roughly similar in the current study (49% increase) compared to the earlier study (57% increase). Thus, prior AICAR treatment has consistently resulted in a substantial increase in glucose uptake by insulin-stimulated muscles from old rats.

Consistent with our previous study, AICAR caused a substantial increase AMPK^Thr172^ phosphorylation in the skeletal muscle from old rats. AICAR also resulted in elevated phosphorylation of ACC^Ser79^, an ACC phosphosite that is frequently used as a surrogate marker for elevated AMPK activity (Hardie 1992; Scott et al. 2002). AICAR also increased the phosphorylation of other AMPK substrates, AS160^Ser704^ and TBC1D1^Ser237^. Muscle γ3-AMPK activity was substantially elevated both immediately after AICAR treatment and more than 3 h after AICAR incubation. However, AICAR did not result in elevated phosphorylation of HMGCR^Ser872^ (an AMPK substrate). The absence of a uniform AICAR effect on the phosphorylation of all AMPK substrates may be related to the fact that, in addition to kinase activation, the phosphorylation status of a specific protein is subject to other regulatory factors, e.g., co-localization of the kinase and substrate, and substrate dephosphorylation by protein phosphatases.

Prior AICAR treatment did not amplify proximal insulin signaling at the level of Akt phosphorylation. This observation is consistent with earlier studies in muscles from young mice (Kjobsted et al. 2015; Jørgensen et al. 2018), young rats (Fisher et al. 2002), and old rats (Oki et al. 2018). AICAR treatment also led to enhanced phosphorylation of AS160 on Thr642 (an Akt phosphosite) and Ser704 (an AMPK phosphosite) in insulin-stimulated muscles. Previous research has reported greater phosphorylation on one or both of these sites in muscles from young mice (Kjobsted et al. 2015; Jørgensen et al. 2018). There is evidence that AMPK-mediated phosphorylation of Ser704 may favor greater Thr642 phosphorylation (Kjobsted et al. 2015). Elimination of AS160 expression in young AS160-KO mice prevented the enhanced insulin-stimulated glucose uptake observed after incubation with AICAR (Kjobsted et al. 2019). In this context, it is reasonable to suspect that AS160 contributed to the effect of AICAR on glucose uptake observed in muscles from old rats in the current study.

AICAR led to greater TBC1D1 phosphorylation on Ser237, which is an AMPK phosphosite (Espelage et al. 2020). Similar results have been reported for muscles from young mice (Vichaiwong et al. 2010; Kjobsted et al. 2015; Jørgensen et al. 2018), but muscles from old animals had not been previously evaluated. Preventing phosphorylation in young mice with a skeletal muscle-specific knock-in mutation of TBC1D1^Ser231Ala^ (Ser231 in mice corresponds to Ser237) resulted in a partial reduction in AICAR-stimulated glucose uptake at a low AICAR concentration (0.15 mM) (Chen et al. 2017). However, the knock-in mutation did not attenuate glucose uptake in response to 2 mM AICAR (Chen et al. 2017), which is the AICAR concentration used in the current study. As expected, phosphorylation of this AMPK phosphosite was unaffected by insulin in muscles of old rats. Neither AICAR nor insulin resulted in greater TBC1D1 phosphorylation on Thr590, which is an Akt phosphosite (Espelage et al. 2020). Some studies have reported that a pharmacologic insulin dose can enhance Thr590 phosphorylation in muscles from young mice (Pehmoller et al. 2009; Vichaiwong et al. 2010). Conversely, an insulin concentration similar to the dose used in the current study failed to increase Thr590 phosphorylation in muscles from either lean or obese middle-aged humans (Middelbeek et al. 2013). Furthermore, insulin-stimulated GLUT4 translocation is not attenuated in skeletal muscle from TBC1D1-KO rats (Whitfield et al. 2017). Although a role for TBC1D1 remains possible, it seems unlikely that the AICAR effect on glucose uptake by the muscles from old rats was entirely attributable to TBC1D1.

Membrane cholesterol was 15% lower for the muscles incubated with AICAR, and this outcome was accompanied by a 49% AICAR-related increase in glucose uptake by insulin-stimulated muscles.

These results are similar to the AICAR-related decrease (~10%) in membrane cholesterol and AICAR-related increase (~50%) in plasma membrane GLUT4 content in insulin-stimulated L6 cells (Habegger et al. 2012a). These findings suggest that the magnitude of AICAR-related change in membrane cholesterol in muscles from old rats might have been sufficient to play a role in the increased glucose uptake. The lack of an AICAR-effect on the phosphorylation of either of the enzymes that can modulate membrane cholesterol content (HMGCR^Ser872^, an AMPK substrate, and ABCA1^Ser2054^, a protein kinase A substrate) indicates that another mechanism was likely responsible for the reduction in membrane cholesterol. However, it is possible that AICAR caused a transient increase phosphorylation of HMGCR^Ser872^ and (or) ABCA1^Ser2054^ that had reversed when the muscles were sampled, more than 3 h after being incubated with AICAR.

Most mammalian cells, including skeletal muscle cells, cannot catabolize cholesterol, and excess cholesterol is either expelled from the cell via transporter proteins or stored as cholesteryl esters in lipid droplets (Luo et al. 2020). While ABCA1 is the cholesterol transporter protein that has been most widely studied in skeletal muscle, mRNA expression of another cholesterol exporter, ABCG1 (ATP binding cassette transporter G1), has also been detected in skeletal muscle (Myers et al. 2006; Cheung et al. 2017; Morgan et al. 2020). AMPK can enhance ABCG1 mRNA and protein expression and cholesterol efflux from macrophages (Li et al. 2010), but evidence is lacking about ABCG1’s protein abundance, regulation, and relative contribution to cholesterol efflux in skeletal muscle. In addition, the possibility that the AICAR-induced decline in membrane cholesterol might be secondary to greater cholesterol esterification and storage in skeletal muscle lipid droplets remains to be evaluated.

Several novel results in the current study advanced knowledge related to the mechanisms underlying improved glucose uptake in skeletal muscle of old rats after brief treatment with AICAR. The most striking new observation was that AICAR treatment led to a marked increase in γ3-AMPK activity, and this increase was sustained more than 3 h after the exposure to AICAR. The magnitude and duration of this effect in the muscle of old rats, taken together with the strong evidence from young mice that γ3-AMPK is crucial for long-lasting effects on glucose uptake after transient AICAR incubation (Kjobsted et al. 2015), supports the idea that γ3-AMPK plays a role in the elevated glucose uptake in AICAR-treated muscles from old rats. It has been widely recognized that the highly selective expression of γ3-AMPK in skeletal muscle offers an opportunity to create a compound with selective action on skeletal muscle with reduced chance of unwanted side-effects in other tissues (Mahlapuu et al. 2004; Kjobsted et al. 2018; Steinberg and Carling 2019; Rhein et al. 2021). Another intriguing and novel result of the current study was the AICAR-induced decrement in membrane cholesterol of skeletal muscle from old rats.

It will be important for future research to determine if γ3-AMPK has a role in the regulation of membrane cholesterol content and to directly test the extent to which the AICAR-induced decrement in membrane cholesterol contributed to elevated glucose uptake. The role of γ3-AMPK could be evaluated using genetically modified mouse models that lack γ3-AMPK expression (Barnes et al. 2004; Rhein et al. 2021). The extent to which a decline in membrane cholesterol plays a role in enhanced glucose uptake could be assessed using the cholesterol-depleting chemical methyl-β-cyclodextrin (MβCD). Previous research demonstrated that MβCD can lower membrane cholesterol in cultured mouse myofibers and palmitate-treated L6 muscle cells (Habegger et al. 2012b; Llanos et al. 2015). Incubating AICAR-treated L6 muscle cells with MβCD that was complexed with cholesterol (MβCD-cholesterol), which can be used to replenish membrane cholesterol, eliminated the AICAR-mediated decrease in membrane cholesterol (Habegger et al. 2012a). MβCD-cholesterol could be used to eliminate the AICAR-induced decrement in skeletal muscle membrane cholesterol content to test if reduced membrane cholesterol is necessary for AICAR’s effect on glucose uptake.

## Data Availability

All relevant data are within the paper.
